# Effect on HIV-1 viral replication capacity of DTG-resistance mutations in NRTI/NNRTI resistant viruses

**DOI:** 10.1186/s12977-016-0265-x

**Published:** 2016-04-30

**Authors:** Hanh T. Pham, Thibault Mesplède, Mark A. Wainberg

**Affiliations:** McGill University AIDS Centre, Lady Davis Institute for Medical Research, Jewish General Hospital, 3755 Ch. Côte-Ste-Catherine, Montreal, QC H3T 1E2 Canada; Division of Experimental Medicine, Faculty of Medicine, McGill University, Montreal, QC Canada; Department of Microbiology and Immunology, Faculty of Medicine, McGill University, Montreal, QC Canada

**Keywords:** HIV-1, Integrase, Viral replication, Integrase inhibitors, R263K, H51Y, Reverse transcriptase inhibitors, K65R, L74V, K103N, E138K and M184V/I

## Abstract

**Background:**

Recommended regimens for HIV-positive individuals include the co-administration of dolutegravir (DTG) with two reverse transcriptase inhibitors (RTIs). Although rare, emerging resistance against DTG is often associated with the R263K substitution in integrase. In-vitro-selected R263K was associated with impaired viral replication capacity, DNA integration, and integrase strand-transfer activity, especially when accompanied by the secondary mutation H51Y. Given the reduced fitness of RTI-resistant viruses, we investigated potential impacts on viral replication of combining R263K and H51Y/R263K with major RTI-resistance substitutions including K65R, L74V, K103N, E138K, and M184I/V.

**Results:**

We combined the R263K or H51Y/R263K with RTI-resistance mutations into the proviral plasmid pNL4.3 and measured the resulting viral infectiousness, replication capacity, and ability to integrate viral DNA into host cells. Infectiousness was determined by luciferase assay in TZM-bl cells. Replicative capacity was monitored over 7 days and viral DNA integration was studied by real-time *Alu*-qPCR in PM1 cells. We found that viral infectiousness, replication capacities and integration levels were greatly reduced in triple mutants, i.e. H51Y/R263K plus a RT mutation, and moderately reduced in double mutants, i.e. R263K plus a RT mutation, compared to wild-type and single RT-mutant viruses.

**Conclusions:**

Our findings help to explain the absence of RTI mutations in individuals who experienced DTG-treatment failure.

## Background

Current antiretroviral (ARV) drugs for treatment of human immunodeficiency virus (HIV) infection include entry inhibitors, protease inhibitors (PIs), nucleoside/non-nucleoside reverse-transcriptase inhibitors (NRTIs/NNRTIs) and integrase strand-transfer inhibitors (INSTIs). In the later class, dolutegravir (DTG) is the most potent drug and can be used without a boosting agent [1]. Unlike raltegravir (RAL) and elvitegravir (EVG), DTG has a higher barrier to resistance, and minimal drug–drug interactions [1, 2]. First-line treatment failure in patients receiving a once daily dose of 50 mg of DTG seems mainly due to non-adherence. Emergence of drug resistance against DTG has not been observed after first-line therapy [3–5]. In second-line treatment of INSTI-naïve patients, a R263K mutation commonly emerged but this substitution causes only about a twofold change in IC_50_, suggesting that the impact on DTG susceptibility is very low. The absence of clinical impact of the R263K mutation has meant that patients harbouring this substitution might be able to remain on DTG therapy [1, 6].

We previously showed that R263K-containing viruses possess modestly reduced viral replication capacity (about 70 % compared to WT) [7, 8]. The R263K substitution was also selected in vitro, and this was followed by the appearance of such secondary substitutions as H51Y, M50I, and E138K [7–10]. However, none of the latter could compensate for the loss in replicative capacity conferred by R263K and, in fact, had the effect of further decreasing viral replication, integrase strand-transfer activity and levels of integrated DNA, even though the addition of H51Y to R263K increased resistance to DTG to about sevenfold, as measured by the PhenoSense^®^ Integrase Replication Assay (7–10). Similar impacts on viral replicative capacity and DTG resistance in tissue culture and biochemical assays were observed when the E138K or M50I substitutions were introduced into R263K viruses. The H51Y/R263K and M50I/R263K viruses may confer similar levels of DTG resistance (about sevenfold resistance against DTG as measured in TZM-bl cells by luciferase assay) while the level of resistance is only about 4.3-fold for the E138K/R263K virus [7, 8, 10].

This is in contrast to the RAL and EVG resistance pathways, in which secondary mutations commonly restored viral replication capacity while, at the same time, adding to the level of drug resistance conferred by initially selected mutations [11–13]. The absence of compensatory secondary mutations for R263K is also consistent with the observation that many major RAL- and EVG-resistance substitutions such as G140S, Q148R, E92Q, N155H and Y143R are incompatible with the simultaneous presence of R263K in terms of both integrase strand-transfer activity and viral replication capacity [14]. In addition, the emergence of R263K as a secondary substitution has been documented in tissue culture under EVG selection pressure as well as in a Phase two clinical trial of the antiviral activity of EVG [15]. More recently, we have shown that the T66I/R263K combination severely impaired both integrase strand-transfer activity and viral replicative capacity. The T66I/R263K virus was slightly more susceptible to DTG when viruses containing R263K alone were studied but highly resistant to EVG, which explains the emergence of this combination with EVG but not DTG [15].

DTG is commonly used in treatment together with abacavir/lamivudine (ABC/3TC) or tenofovir/emtricitabine (TDF/FTC) in ARV-naïve patients. However, the combined effects of DTG- and RT-resistance mutations have not been well studied until now, although it is known that the combination of M184I/V and R263K in the same virus further decreased viral replication compared to either mutation alone and no DTG or lamivudine cross-class resistance was observed [16]. Now, we have studied the effect of R263K or H51Y-R263K in the presence of other clinically relevant RT mutations, i.e. K65R, L74V, K103N, E138K, and M184I/V. Of the above, K103N is a major mutation associated with resistance to efavirenz and etravirine; both NNRTIs are known to reduce plasma levels of DTG, thereby limiting their use in co-administration with DTG [17, 18]. The E138K substitution in RT has been selected in patients treated with rilpivirine and efavirenz and has resulted in cross-resistance to NNRTIs. Each of K65R, L74V and M184I/V are major NRTI-related mutations [17, 18]. Tenofovir, ABC, lamivudine and didanosine can select for K65R, while lamivudine and emtricitabine select for M184I/V. In the phase 3 102 and 103 clinical studies of a once daily pill of EVG/cobicistat/FTC/TFV, the K65R and M184I/V substitutions were detected, separately or in combination, before the emergence of IN mutations in some patients undergoing treatment failure [19, 20]. L74V has been selected by didanosine and ABC and can confer high-level resistance against these drugs [17, 18].

## Results

### The addition of DTG-resistance substitutions reduces viral infectiousness in viruses containing the K65R, L74V, K103N, E138K or M184V/I reverse transcriptase mutations

**Fig. 1:**
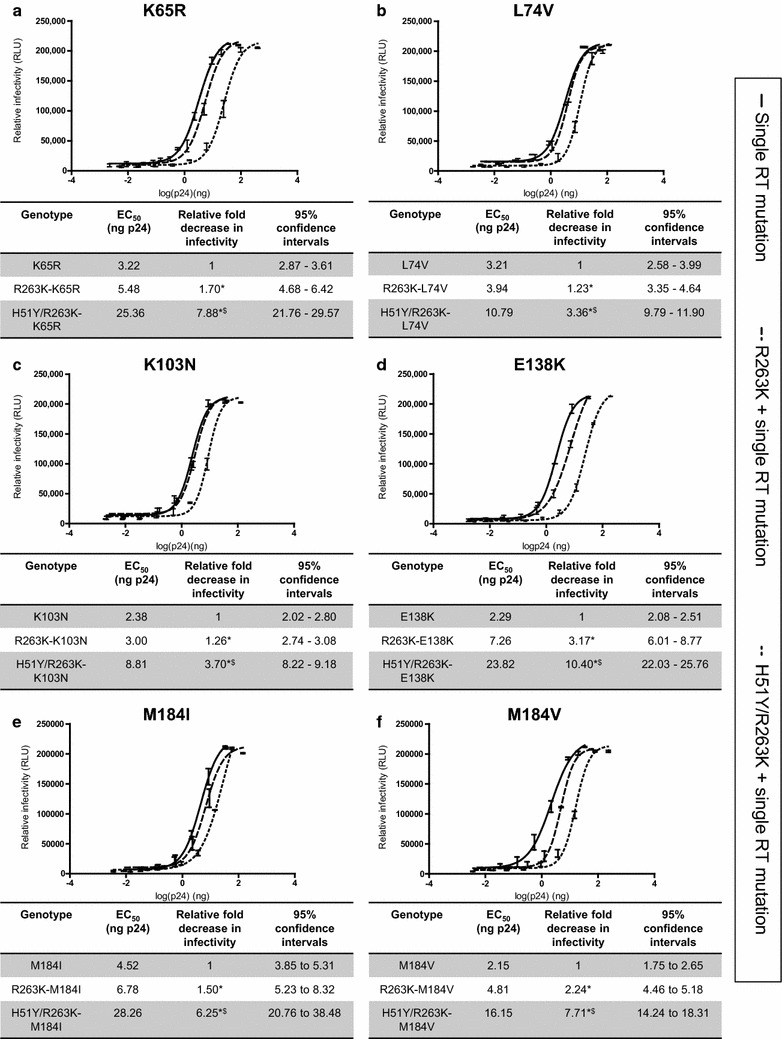
Effects of R263K and H51Y/R263K in combination with different RTI substitutions on viral infectivity in TZM-bl reporter cells. Expressed luciferase levels were measured at 48 h.p.i. and normalized to relative p24 levels. Best-fit curves and data for EC_50_ values, 95 % confidence intervals and fold changes (FC) relative to single RT mutations alone are showed in the *insets*: **a** K65R series including NL4.3_K65R_, NL4.3_R263K-K65R_, and NL4.3_H51Y/R263K-K65R_. **b** L74V series including NL4.3_L74V_, NL4.3_R263K-L74V_, and NL4.3_H51Y/R263K-L74V_. **c** K103N series including NL4.3_K103N_, NL4.3_R263K-K103N_, and NL4.3_H51Y/R263K-K103N_. **d** E138K series including NL4.3_E138K_, NL4.3_R263K-E138K_, and NL4.3_H51Y/R263K-E138K_. **e** M184I series including NL4.3_M184I_, NL4.3_R263K-M184I_, and NL4.3_H51Y/R263K-M184I_. **f** M184V series including NL4.3_M184V_, NL4.3_R263K-M184V_, and NL4.3_H51Y/R263K-M184V_. The FC value of each singly mutated RT virus was arbitrarily set at 1. *Error bars* indicate mean ± standard deviation (SD). *Asterisk* and *dollar sign* indicate statistically significant difference from single RT mutants alone and from the R263K-containing viruses, respectively (Student’s t test, P < 0.05)

As previously hypothesized, the fitness cost of the H5Y/R263K combination may have a benefit for patients under DTG treatment in terms of viral load. Given the current co-administration of DTG with RTIs for HIV treatment, we wanted to determine whether the R263K and H51Y/R263K mutations may have similar effects on viruses that contained mutations in RT that confer resistance to RTIs. First, we created chimeric HIVs having R263K or both H51Y/R263K in IN together with any of a number of major RTI-resistance substitutions, i.e. K65R, L74V, K103N, E138K, or M184I/V. We then performed single round infections in TZM-bl cells and measured levels of luciferase expression after 48 h.p.i.; these levels were normalized on the basis of p24 expression. Levels of viral infectivity were determined for each of the various DTG-resistant and RTI-resistant viruses and those containing only a single RT mutation in the absence of drugs. Our results show that the addition of R263K to the K65R-, L74V-, K103N-, E138K-, or M184I/V-harbouring viruses resulted in further moderate decreases in viral infectivity ranging from 1.23-fold to 3.17-fold (P < 0.05), depending on the RT backbone (Fig. 1a–f). In viruses carrying K65R, E138K, or M184V, the additional presence of R263K in IN coding sequence caused a further impact on viral replication than was associated with any of these RT mutations on their own. These data are consistent with previous findings that reported that the presence of R263K in combination with M184I/V resulted in a further 1.8-fold decrease in replication capacity compared to M184I or M184V alone [16]. The most negative impact observed was when R263K was combined with E138K, yielding a 3.2-fold decrease in infectiousness compared to E138K alone (Fig. 1d), whereas only slight decreases, about 1.2-fold were observed for R263K-L74V (Fig. 1b) and 1.3-fold for R263K-K103N (Fig. 1c) viruses compared to L74V and K103N alone, respectively.

The RT mutant viruses that also carried the integrase double mutations, i.e. H51Y/R263K were very impaired in replication (P < 0.05), i.e. a 3.36-fold–10.4-fold decrease compared to the single RT mutations alone (Fig. 1a–f). The order of diminished infectiousness with H51Y/R263K was as follows: E138K, relative fold-change (FC) = 10.4 (Fig. 1d), K65R with FC = 7.88 (Fig. 1a), M184V with FC = 7.71 (Fig. 1f), and M184I with FC = 6.25 (Fig. 1e). The addition of double mutations H51Y/R263K to the L74V or K103N viruses also affected viral replication but at lower levels, i.e. 3.36 and 3.70 FC (Fig. 1b, c). R263K on its own together with any one of the RT mutations in the same virus yielded only slight reductions in viral infectivity compared to either mutation alone, and the presence of both H51Y/R263K together with the RT mutations had the most severe consequence on infectiousness. We also conducted RT assays to validate our p24 results and found that a comparison of results from RT and p24 assays yielded similar findings for WT or viruses containing the R263K or H51Y/R263K substitutions (Table 1), but not for viruses containing mutations in the RT sequence (data not shown).

**Table 1:** Comparison of RT and p24 assays for normalization of viral infectivity in TZM-bl cells

Genotype	p24 assay			RT assay		
	EC_50_ (ng p24)	Relative fold decrease in infectivity	95 % Confidence intervals	EC_50_ (cpm)	Relative fold decrease in infectivity	95 % Confidence intervals
WT	3.57	1	2.77–4.58	3270	1	2545–4200
R263K	4.64	1.3*	3.68–5.84	3864	1.2*	3067–4868
H51Y/R263K	27.12	7.6*^,$^	24.65–29.18	22,964	7*^,$^	20,870–25,269

*^,$^ Statistically significant difference from WT and R263K viruses, respectively (Student’s t test, P < 0.05)

### Combining DTG- and RTI-resistance mutations reduces viral replicative capacity

**Fig. 2:**
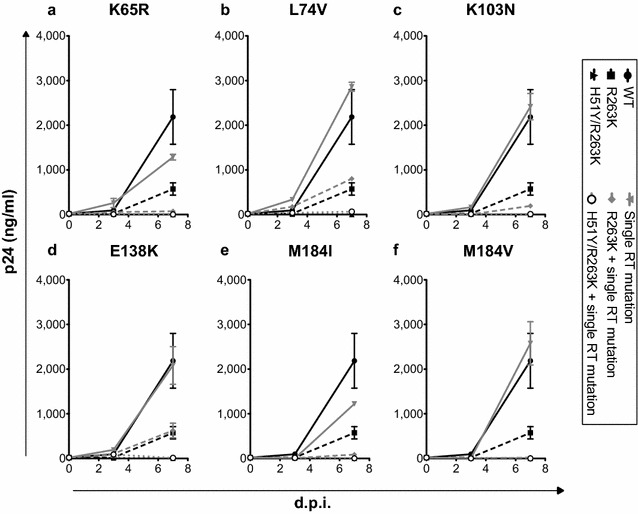
Viral replication kinetics over a 7-day period in PM1 cells. PM1 cells were infected with the same amount of p24 for all viral stocks. Levels of p24 production in cell-free supernatants were monitored at days 3 and 7 p.i. WT, R263K and H51Y/R263K viruses are also presented in each data series from **a** to **f**. **a** K65R series, **b** L74V series, **c** K103N series, **d** E138K series, **e** M184I series, and **f** M184V series. Values were calculated from three independent infections. *Error bars* indicate mean ± SD. *ND* not detectable

Next we infected PM1 cells with our various viruses in the absence of drugs using the same quantity of p24 antigen in each case over a 7-day period and harvested virus-containing cell culture fluids at days 3 and 7 p.i. for p24 measurements. At day 3 p.i., we obtained detectable levels of p24 with both the WT and single RT mutation viruses, showing that these viruses began to replicate very early; the p24 levels continued to increase over 7 days (Fig. 2a–f). In contrast, none of the R263K-containing viruses produced detectable p24 in our assay until day 7 p.i. (Fig. 2a–f). However, the viruses that contained H51Y/R263K plus a RT mutation or only H51Y/R263K were unable to generate detectable p24 at day 7, suggesting that significant replication had not occurred (Fig. 2a–f). These results from replication kinetics over a 7-day period of infection lend support to the observation that the presence of R263K or H51Y/R263K in the IN sequence together with K65R, L74V, K103N, E138K, or M184V/I in the same virus resulted in slight to significant decreases in viral replication capacity (Fig. 1a–f). In both experiments, the L74V and K103N viruses were the least affected by the presence of H51Y/R263K or R263K.

### Integration capacity of DTG- and RTI-resistant viruses

**Fig. 3:**
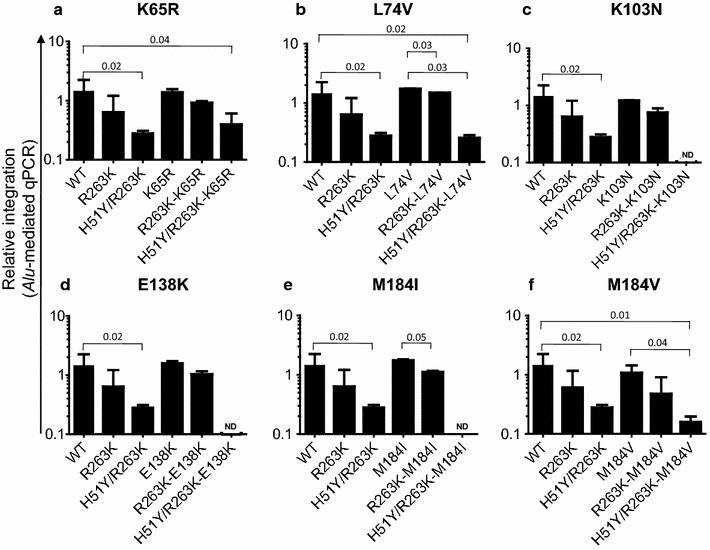
Integrated HIV DNA levels in PM1 cells at day 2 p.i. were measured by *Alu*-mediated qPCR assays (presented on a log scale). *Error bars* indicate mean ± SD. **a** K65R series, **b** L74V series, **c** K103N series, **d** E138K series, **e** M184I series, and **f** M184V series. Only P values with statistic significance were showed. The levels of integration are relative to WT virus at 0 h post infection. *ND* not detectable

We next wished to determine whether the integration efficiency of the viruses containing the various combinations discussed above might be diminished in tissue culture. For each virus, we used the same amount of p24 to infect PM1 cells and harvested infected cells after 2 days. Total DNA was extracted and *Alu*-mediated qPCR was performed to quantify integration levels of HIV-1 DNA into PM1 cells. The results show that all the RTI-resistant viruses that also contained R263K exhibited slightly decreased levels of viral integration capacity, about 0.1 log–0.5 log reduction, compared to the single RT mutation viruses and WT (Fig. 3a–f). However, these differences were not statistically significant (all P values >0.05, Fig. 3a, c, d, f), except for L74V versus R263K-L74V (P = 0.03, Fig. 3b) and M184I versus R263K-M184I (P = 0.05, Fig. 3e). Results from the *Alu*-mediated qPCR studies of viruses containing H51Y/R263K showed low levels of integrated DNA in cases of these viruses H51Y/R263K-K65R, H51Y/R263K-L74V and H51Y/R263K-M184V (Fig. 3a, b, f), or undetectable levels in H51Y/R263K-K103N, H51Y/R263K-E138K and H51Y/R263K-M184I viruses (Fig. 3c–e) in comparison to those of single RT mutants and WT virus (P values with statistical significance are shown in Fig. 3a, b, f), consistent with the viral replication data discussed above [8, 9]. However, this trend did not reach statistical significance for the K65R versus H51Y/R263K-K65R viruses (P = 0.08).

## Discussion

Current HIV treatment guidelines for first line therapy favor the use of an INSTI together with two NRTIs. In treatments based on the use of RAL or EVG, the co-existence of NRTI and INSTI resistance mutations has been reported in patients experiencing therapeutic failure [19–21]. Most commonly, mutations at N155H and Q148H/R have been observed in combination with M184I/V or less often with K65R. In some of these cases, both K65R and M184V/I have co-emerged with integrase mutations. Fitness defects were also observed for viruses containing these RAL/EVG-resistance mutations together with RT mutations in vitro in the absence of drugs [22, 23]. In contrast, neither DTG nor NRTI resistance has been reported until now in treatment-naïve patients who received DTG plus NRTI treatment. Given that the R263K substitution has been reported in some INSTI-naïve patients in the SAILING study and in tissue culture selections, we aimed to investigate the effects of R263K and its secondary mutation H51Y together with major RTI-resistance substitutions at positions K65R, L74V, K103N, E138K, and M184I/V, in terms of viral replication and integration capacity in the absence of drugs.

First, our study confirmed previous observations on the decrease of viral replication in viruses containing both M184I/V plus R263K [16]. We also confirmed the deficits caused by R263K together with other RTI mutations, i.e. K65R, L74V, K103N, and E138K. However, small changes in the results of infectivity assays may or may not result in differences in infectivity in vivo. Compared to WT virus, the R263K substitution as well as some other major mutations E92Q, Q148R and N155H, reported in the RAL- and/or EVG-resistance pathways, can emerge from a single nucleotide change in most HIV-1 strains. Each of these mutations led to a decrease in viral replicative fitness (less than sevenfold) and integrase strand-transfer activity (less than threefold). However, the levels of resistance associated with these various substitutions for different INSTIs varied considerably. For example, N155H virus caused sixfold and fivefold decreases in susceptibility to RAL and EVG and E92Q conferred more than a 50-fold decrease in EVG susceptibility. Similar sixfold–sevenfold levels of resistance to RAL and EVG have been reported for Q148R virus [14]. In contrast, the common M184V mutation develops rapidly in patients failing lamivudine (3TC) therapy. Although this mutation alone confers up to 100-fold–1000-fold less susceptibility to 3TC, regimens containing this drug have been shown to maintain efficacy in the treatment of HIV-1, perhaps because the M184V mutation is associated with a loss of replicative fitness and increased fidelity of RT activity [24]. Nevertheless, the combination of M184V with K65R has caused virological failures in patients receiving regimens containing two N(t)RTIs, 3TC and tenofovir [25].

On the other hand, R263K is more commonly detected in HIV-positive individuals who experience treatment failure with DTG even though this substitution only modestly decreases DTG susceptibility (about twofold) as well as HIV-1 replication capacity. This raises the question as to why this substitution is not more commonly observed in clinical settings since its effect on viral fitness is insufficient on its own to explain its absence in most individuals failing DTG-based regimens. One possibility is that viruses that contain R263K and other resistance mutations, e.g. H51Y, are so impaired in replication capacity that they cannot be detected by ordinary Sanger sequencing. Another possibility is that DTG may reach anatomical compartments that are not susceptible to other inhibitors, effectively preventing the emergence of R263K and other substitutions; this possibility is supported by recent reports of persistent HIV replication under suppressive ARV therapy [1, 26].

A related question is why the R263K substitution that confers low-level resistance to DTG is selected at all. The answer is probably that R263K does confer a higher level of resistance against DTG as a single substitution than does any of the resistance mutations associated with RAL or EVG (1). This is fortuitous for the use of DTG even in patients who are not adherent to therapy, especially because the R263K substitution does not seem to be able to co-exist together with mutations at positions 143 and 148 that are frequently observed in patients who have failed therapy with RAL or EVG (14). Secondary resistance mutations usually follow after primary mutations and can both compensate for the loss of viral replication caused by the initial mutation while also resulting in higher levels of drug resistance [1]. However, no secondary mutation for DTG can compensate in this fashion [7, 8, 10]. We previously demonstrated that the secondary mutation H51Y developed after the emergence of R263K in vitro and caused a higher level of resistance against DTG, about 16-fold in TZM-bl cells and sevenfold in the PhenoSense^®^ Integrase Replication assay. H51Y also has a further detrimental effect on viral replication capacity. The current study also shows that this replication deficit is further exacerbated in the presence of different RT resistance mutations, helping to explain the absence of resistance to both DTG and RTIs in first-line therapy.

K103N is a very common NNRTI mutation that only minimally affects viral replication in the absence of drug pressure [27, 28]. The addition of R263K to K103N caused only a small loss in viral replication capacity compared to K103N alone. L74V is associated with a reduced replicative capacity of HIV-1 of about 11 % compared to WT [29, 30]. In our study, decreased replication was also observed with the R263K-L74V virus and this was greatly exacerbated by the additional presence of H51Y. Similar results were obtained with K103N together with R263K/H51Y, with no detection of p24 at days 3 and 7 p.i. Our data show that differences in viral replication in the presence of different resistance mutation combinations may not only be due to possible additive effects but may also reflect possible interactions between RTI- and DTG-resistance mutations. The addition of R263K to viruses containing M184I or M184V, K65R, and E138K yielded fewer viruses that were replication competent than when only single RT mutations were studied. And these differences were further magnified in the presence of H51Y. Possibly as well, the presence of some triple combination mutations may alter interactions between RT and IN, leading to a loss of viral replication and/or generation of non-viable viruses. In the presence of drug pressure, it may be very difficult for HIV-1 to develop all of these mutations in IN and RT at the same time. These findings are consistent with earlier results that the emergence of M184I was delayed in the presence of R263K under lamivudine drug pressure and that the M184I substitution was detected in the case of the H51Y/R263K virus [31].

Here we also report on the integration capacity of the mutational combinations studied in PM1 cells in comparison to that of WT virus and viruses containing only single RT mutations. Double mutant viruses with RT and R263K integrated less efficiently than either viruses with single RT mutations or WT whereas DNA integration levels in almost all triple mutant viruses were very low or undetectable. Although small differences in integration levels were observed for single RT mutants and double R263K-RT mutants, these were not significant (Student’s t test, P values >0.05), whereas statistical significance was achieved for L74V versus R263K-L74V (Student’s t test, P = 0.03) and M184I versus R263K-M184I (Student’s t test, P = 0.05). In addition, triple mutants bearing the H51Y/R263K-RT mutations resulted in either undetectable integration levels (in cases of H51Y/R263K plus single substitutions K103N, E138K or M184I, Fig. 3c–e) or levels of integration that were statistically inferior to those attained with the single RT mutants (P ≤ 0.05). L74V and M184I are associated with resistance against ABC, which is co-formulated with DTG and lamivudine in a single daily pill; these results suggest that this combination may provide an advantage in regard to pathways leading to drug resistance.

Our integration data, however, did not always directly correlate with the infectivity data (Fig. 1a). For example, H51Y/R263K-K65R triple mutants resulted in appreciable levels of integration that were comparable with those observed with the double mutant R263K-K65R virus (Student’s t test, P = 0.2), whereas the same H51Y/R263K-K65R combination of substitutions attenuated infectivity by 7.8-fold compared to the R263K-K65R combination that only caused a 1.7-fold decrease (Fig. 1a). Although both assays demonstrated a tendency for the triple mutants to be more impaired in both integration and infectivity than the double mutants, some discrepancies in the extent of the impairment as measured in these various assays clearly exist. This may be due to the fact that integration levels were measured using a single viral inoculum whereas the infectivity assays used virus at various titers. In addition, there may be differences between the susceptibilities of different cell lines to becoming infected by the different viruses that were employed. Indeed, integration was necessarily measured in PM1 cells, which support productive HIV-1 replication, whereas the TZM-bl cells used in the infectivity assay do not support multiple cycles of active replication. It is well established that different methods can yield different results in regard to infectivity and resistance assays [8].

Since integration is important for virus to establish latent reservoirs in host cells, we conjecture that the diminution in levels of integration associated with resistance to DTG may have consequences for the size of the viral reservoir. Further studies on the capacity of latency reservoirs to be established in patients with DTG-resistant viruses or in patients treated with DTG will provide important information on long-term DTG treatment efficacy [1].

## Conclusions

DTG- and RTI-containing viruses are associated with significant loss of viral fitness and DNA integration capacity. This could help to explain the successful use of DTG in treatment of HIV in co-administration with two-NRTI backbones ABC/TDF or FTC/TDF. Our results provide additional information to explain the absence of clinically relevant resistance to DTG when used in combination first-line therapy.

## Methods

### Cells and reagents

TZM-bl and 293T cells were cultured in Dulbecco’s Modified Eagle Medium (DMEM). PM1 cells were cultured in Roswell Park Memorial Institute Medium (RPMI). Cell culture media were supplemented with 10 % fetal bovine serum (FBS), 2 mM l-glutamine, 50 U/ml penicillin, and 50 µg/ml streptomycin and incubated at 37 °C under 5 % CO_2_.

### Generation of infectious HIV-1 stocks

pNL4.3, pNL43_R263K_, pNL4.3_H51Y/R263K_, pNL4.3_M184I_, pNL4.3_M184V_, pNL4.3_R263K-M184I_, pNL4.3_R263K-M184V_, pNL4.3_K65R_, pNL4.3_L74V_, pNL4.3_K103N_, and pNL4.3_E138K_ were previously constructed using site-directed mutagenesis [16, 32, 33]. Similar methods were employed to produce these plasmids: pNL4.3_R263K-K65R_, pNL4.3_H51Y/R263K-K65R_, pNL4.3_R263K/L74V_, pNL4.3_H51Y-R263K/L74V_, pNL4.3_R263K-K103N_, pNL4.3_H51Y/R263K-K103N_, pNL4.3_R263K-E138K_, pNL4.3_H51Y/R263K-E138K_, pNL4.3_H51Y/R263K-M184I_, and pNL4.3_H51Y/R263K-M184V_. The primer sequences used for PCR site-directed mutagenesis were previously described [8, 9]. All clones that were generated after transformation were purified and sequenced to confirm the presence of expected mutations and have no contamination. To produce infectious homogenous viruses, 12.5 µg of each plasmid DNA purified from maxi cultures that were themselves initiated from a single bacterial clone was transfected into 293T cells using Lipofectamine 2000 (Invitrogen). Fresh medium was added at 4 h.p.i. Culture supernatants containing single cycle HIV virions were collected at 48 h post transfection and passed through a 0.45-µm filter. Virus stocks were aliquoted into small volumes of 500 µl and stored at −80 °C. Freshly thawed virus stocks were used for every experiment and virus stocks left after each usage were discarded and never used again. Quantification of viruses was performed using p24 and RT assays as described previously [8, 9].

### HIV-1 infectivity assay

HIV-1 infectivity studies were performed by luciferase assay and by short-term infection of TZM-bl reporter cells. Thirty thousand TZM-bl cells/well were infected with a series of diluted viruses in a 96-well flat-bottom plate. Cells were lysed with Promega lysis buffer at 48 h.p.i. Luciferase levels were measured using the Luciferase Assay System (Promega, Madison, WI, USA). Experiments were performed in triplicate and repeated twice. Fold changes in the decreases of infectivity (FC) were represented as relative EC_50_, which is the amount of virus (previously quantified using p24 and RT assays) needed for TZM-bl cells to produce half of the maximal level of luciferase in an infection.

### Replication capacity assay

Thirty thousand PM1 cells were infected with 18 ng of p24 for each virus. Briefly, cells were incubated with viruses for 1 h at 37 °C. After incubation, cells were washed to remove unbound viruses and seeded in 96-well plates. Cell-free medium containing viruses was withdrawn for p24 measurements at days 3 and 7 p.i. The same volumes of fresh medium and new cells were added at each time point. Experiments were done in triplicate and each study of RT mutated viruses was performed separately.

### Determination of integrated-HIV DNA

A total of 2 × 10^5^ PM1 cells were infected with 125 ng p24. Cellular DNA was extracted at 48 h.p.i., using a DNeasy blood and tissue kit (Qiagene). A two-step *Alu*-gag PCR was performed as previously described [34]. The first-round PCR was performed with a 65 ng DNA sample in a 25 µl reaction with primers *Alu*-F: 5′-GCCTCCCAAAGTGCTGGGATTACAG-3′ and *Gag*-R: 5′-GTTCCTGCTATGTCACTTCC-3′. The first cycle conditions were: 95 °C for 2 min, followed by 40 cycles of 95 °C for 15 s, 50 °C for 15 s, 72 °C for 3 min 30 s, and final elongation was at 72 °C for 5 min. The second-nested PCR was performed on a Corbett Rotor-Gene 6000 thermocycler, using Platinum quantitative PCR (qPCR) SuperMix-UDG (Invitrogen) with 5 µl of first-round PCR product in a 20 µl reaction. The primer sequences were LTR-F: TTAAGCCTCAATAAAGCTTGCC and LTR-R: GTTCGGGCGCCACTGCTAGA with the following cycle conditions: 50 °C for 2 min, 95 °C for 2 min, 60 cycles of 95 °C for 10 s, 60 °C for 10 s and 72 °C for 45 s. All samples were normalized in terms of β-globin content. The levels of integration are relative to WT virus at 0 h post infection.

### Statistical analyses

Each experiment was repeated twice using three replicate samples each time. Data analyses including linear and non-linear regressions, and determinations of EC_50_, standard deviation, 95 % confidence intervals, and relative FC were calculated using GraphPad Prism 5.0 software. We used two-tailed Student’s t test with a 0.05 cut-off point to compare the best-fit values or means of each pair of different data sets.
